# The interplay of physical activity, sleep, and chronotype on physical and mental health in Italian university students: a moderated mediation analysis

**DOI:** 10.1186/s12889-025-25951-8

**Published:** 2025-12-19

**Authors:** Lucia Castelli, Letizia Galasso, Marta Borrelli, Fabio Esposito, Andrea Caumo, Giovanni Michielon, Eliana Roveda, Angela Montaruli

**Affiliations:** Department of Biomedical Sciences for Health, University of Milan, Via Giuseppe Colombo 71, Milan, 20133 Italy

**Keywords:** Circadian typology, Morningness/eveningness, Sports science students, Self-perceived sleep, Regular exercise, Quality of life

## Abstract

**Background:**

Physical and mental health are receiving increasing attention among university students. This study aims to evaluate the relationship between physical activity level, chronotype, and sleep quality in influencing physical and mental health in a sample of 691 active Italian university students.

**Methods:**

Participants completed the GSL-TPAQ, MEQ, PSQI, and SF-12 questionnaires and were divided into three categories based on physical activity level (low, medium, high). A three-way ANCOVA assessed the main effects of physical activity, chronotype, and sleep quality on physical and mental health. A moderated mediation model tested whether sleep quality mediated the relationship between chronotype and health, and whether physical activity moderated the effect of sleep quality on health.

**Results:**

A three-way ANCOVA revealed that high physical activity, morningness, and good sleep quality were associated with better mental health. Specifically, students with high physical activity reported higher mental health (43.23 ± 8.97 a.u.) than those with low activity (40.65 ± 10.21 a.u.; *p* < 0.05). Morning-types reported better mental health (43.6 ± 9 a.u.) compared to evening-types (39.08 ± 10.05 a.u.; *p* < 0.001), and good sleepers scored significantly higher on mental health (43.64 ± 9.04 a.u.) than bad sleepers (37.7 ± 9.68 a.u.; *p* < 0.001). Good sleepers also had better physical health (52.55 ± 5.32 a.u.) than bad sleepers (50.14 ± 5.97 a.u.; *p* < 0.001). A moderated mediation model confirmed that sleep quality mediated the relationship between chronotype and mental health (indirect effect: B = –0.08, 95% CI [–0.12, –0.04]), with morningness associated with better sleep, and in turn, better mental health. Physical activity significantly moderated this effect: the index of moderated mediation was significant for medium activity (Index = –0.04, 95% CI [–0.08, –0.01]) and high activity (Index = –0.04, 95% CI [–0.07, –0.01]), suggesting that individuals with medium and high activity levels benefit most from the protective role of sleep in the chronotype–mental health pathway.

**Conclusion:**

These findings underscore the need to promote both good sleep hygiene and regular, sustained physical activity to support mental well-being among university students, especially those with an evening chronotype or bad sleep quality.

## Background

University students represent a distinct and dynamic demographic, often drawing the attention of researchers due to the unique health challenges they face [1–3]. A strong foundation of health is closely linked to academic success, enabling students to pursue their goals more effectively, engage enthusiastically with their studies, and enjoy a more fulfilling university experience [4, 5]. Importantly, the university years serve as a formative period during which healthy habits—once established—can influence well-being well into adulthood and later life [6, 7].

Among the many lifestyle factors affecting student health, physical activity, chronotype, and sleep patterns stand out [8–14]. Alongside diet and academic workload, these elements play a pivotal role in shaping both physical and mental well-being [8–14].

As with other populations, engaging in regular and sustained physical activity is associated with improved health outcomes and a better quality of life [13, 15, 16]. An active lifestyle among university students is often positively correlated with overall wellness [12, 17, 18], and a growing body of research is focused on understanding and overcoming the barriers that limit physical activity in this group [19].

Chronotype is an individual’s natural preference for activity and rest periods based on circadian rhythms [20, 21]. They are governed by the suprachiasmatic nucleus (SCN) in the hypothalamus, which receives light–dark signals from the retina and aligns them with sleep–wake timing. Peripheral clocks throughout the body are synchronized by the SCN [22]. This system, influenced by genetics, defines individual chronotypes, which are increasingly being examined in relation to student health [23]. Typically classified as morning-type, evening-type, or neither-type, chronotype significantly influences daily functioning. Morning-types (M-types), who prefer early wake times and are more active in the first part of the day, often find themselves better aligned with academic schedules [20, 21, 24]. In contrast, evening-types (E-types) tend to experience a mismatch between their natural rhythm and institutional demands [20, 21], which may contribute to a worse sleep quality [25, 26] and a heightened risk of both physical and mental health issues [9, 23].

Sleep quality and duration are equally vital to student health [27, 28]. Bad sleep is a well-documented risk factor for numerous health problems among university students [27, 28]. However, maintaining healthy sleep habits can be particularly challenging during these years, which are frequently marked by late-night social activities and intensive study sessions—both of which can significantly reduce overall sleep time [29].

These variables may interact in complex ways to influence health outcomes, with several studies highlighting their interrelationships across different populations and contexts, often through mediation and moderation analyses [30–33]. Among university students, Roeser and colleagues (2012) identified self-perceived sleep quality as a mediator in the link between chronotype and stress. Their findings suggest that E-types are more prone to bad sleep quality, which in turn heightens their vulnerability to stress and emotional dysregulation [8]. Likewise, research by Dickinson and colleagues (2018) and Bakotić and colleagues (2017) reported that E-types are more likely to experience anxiety [34] and depression [34, 35] with these associations being mediated by various indicators of bad sleep quality [34]. However, studies examining mediation or moderation models that incorporate physical activity rarely focus on the health of university students and often overlook the roles of chronotype and sleep as contributing factors [36, 37].

Given that most previous studies have not simultaneously examined physical activity, chronotype, and sleep within a single model—particularly in relation to the mental health of university students—nor explored the potential interplay among these variables, the present study aims to address this gap. Specifically, we aim to explore how physical activity, chronotype, and sleep interact to influence both physical and mental health in university students. We hypothesize that sleep functions as a mediator in the relationship between chronotype and health, while physical activity may moderate the association between sleep and health outcomes. To test these hypotheses, we developed two moderated mediation models, one using physical health as the dependent variable and the other using mental health as the dependent variable. These models allow us to investigate the mediating role of sleep and the moderating influence of physical activity in the overall relationship structure.

## Materials and methods

### Study design

This research was conducted within the framework of the SINCRONA project, an acronym in Italian that stands for Sleep (Sonno), Inactivity (Inattività), Chronotype (Cronotipo), and Diet (Alimentazione). Data were gathered via an online survey administered in November 2022. The survey included general information (such as age, height, weight, university enrollment year, education level, and origin), along with four standardized questionnaires, described in detail below.

Students were approached during in-person lectures to clarify the purpose of the study and the procedures for data collection. At the end of the presentation, they were provided with a link to the online survey hosted on EU Survey. EU Survey is an online platform authorized by the University of Milan for conducting this type of research. Participation was voluntary. Before completing the survey, potential participants were again provided with a description of the project, asked to give their consent, and informed about data privacy conditions. They were then able to complete the survey. After completion, the data were available in an Excel file, accessible only to the authorized researchers of the project.

### Participants

Participants (*n* = 691; age: 20.11 ± 1.22 years; males: 506, 73.2%) were recruited from second and third-year bachelor’s students enrolled in the Sports Science program at the University of Milan, a population characterized by a consistently high level of physical activity.

Inclusion criteria were:

- No significant changes in physical activity levels or sleep habits within the two months prior to data collection;
- No use of medications, recreational drugs, or other substances known to affect sleep;
- Not pregnant at the time of the study (self-reported);
- No recent acute or chronic health conditions in the past two months that could interfere with sleep or physical activity;
- Absence of any diagnosed medical conditions.

Exclusion criteria were defined as the presence of at least one of the aforementioned conditions.

### Questionnaires

#### Godin-Shepard Leisure-Time Physical Activity Questionnaire (GSL-TPAQ)

The questionnaire by Godin & Shephard [38, 39] was used to assess physical activity during the previous week. Physical activity is assessed by asking the number of times per week of three physical activity intensities (strenuous, moderate, light), assigned different METs values (strenuous = 9 METs, moderate = 5 METs, light = 3 METs). Based on the original categorization of the questionnaire, all participants were classified as active. Therefore, based on the final score, expressed as Leisure Score Index (LSI), participants were stratified into three tertiles: LSI 14 - ≤49: low physical activity tertile (low); LSI > 49 - ≤68: medium physical activity tertile (medium); LSI > 68: high physical activity tertile (high). In the current study, the Cronbach α for the GSL-TPAQ was 0.6.

#### Morningness-Eveningness Questionnaire (MEQ)

Chronotype was assessed with the Italian version of the Morningness-Eveningness Questionnaire (MEQ) [20, 40]. Based on the final score (expressed in arbitrary units - a.u.), participants were classified as E-types (16–41 a.u.), N-types (42–58 a.u.), or M-types (59–86 a.u.). In the current study, the Cronbach α for the MEQ was 0.8.

#### Pittsburgh sleep quality index (PSQI)

This questionnaire evaluated participants’ sleep quality over the previous 30 days [41]. The total score ranges from 0 to 21 arbitrary units (a.u.), where lower values reflect better self-perceived sleep quality. A threshold score of 5 a.u. was used to distinguish between good and bad sleepers. We administered the Italian version of the questionnaire [42]. In this study, the internal consistency of the PSQI, as measured by Cronbach’s alpha, was 0.6.

#### Short-Form health survey (SF-12)

The Italian version of the 12-item health questionnaire was used to assess both physical and mental health dimensions, specifically the Physical Component Summary (PCS) and the Mental Component Summary (MCS), with higher values indicating a better perceived health status [43, 44]. The SF-12 demonstrated acceptable internal consistency in the present study, with a Cronbach’s alpha of 0.7.

### Statistical analysis

All analyses were conducted using IBM SPSS Statistics for Windows, Version 29 (IBM Corp., Armonk, NY, USA). Moderated mediation analyses were performed using the PROCESS macro for SPSS (version 4.1), developed by Andrew F. Hayes [45].

Statistical significance was set at an alpha level of 0.05, with 95% confidence intervals (CIs). Continuous variables are presented as means and standard deviations (SD), while categorical variables are reported as frequencies and percentages. All analyses were adjusted for age and sex (coded as follows: 0 = female, 1 = male).

The statistical analysis involved three main steps. First, a three-way ANCOVA was conducted to assess the main effects and relative interactions of physical activity (GSL-TPAQ), chronotype (MEQ), and sleep quality (PSQI) on health outcomes measured by the SF-12.

Second, partial Spearman correlation analyses were performed among the continuous variables (MEQ, PSQI, and SF-12) to explore associations and determine which variables were appropriate for inclusion in the moderated mediation models.

**Fig. 1:**
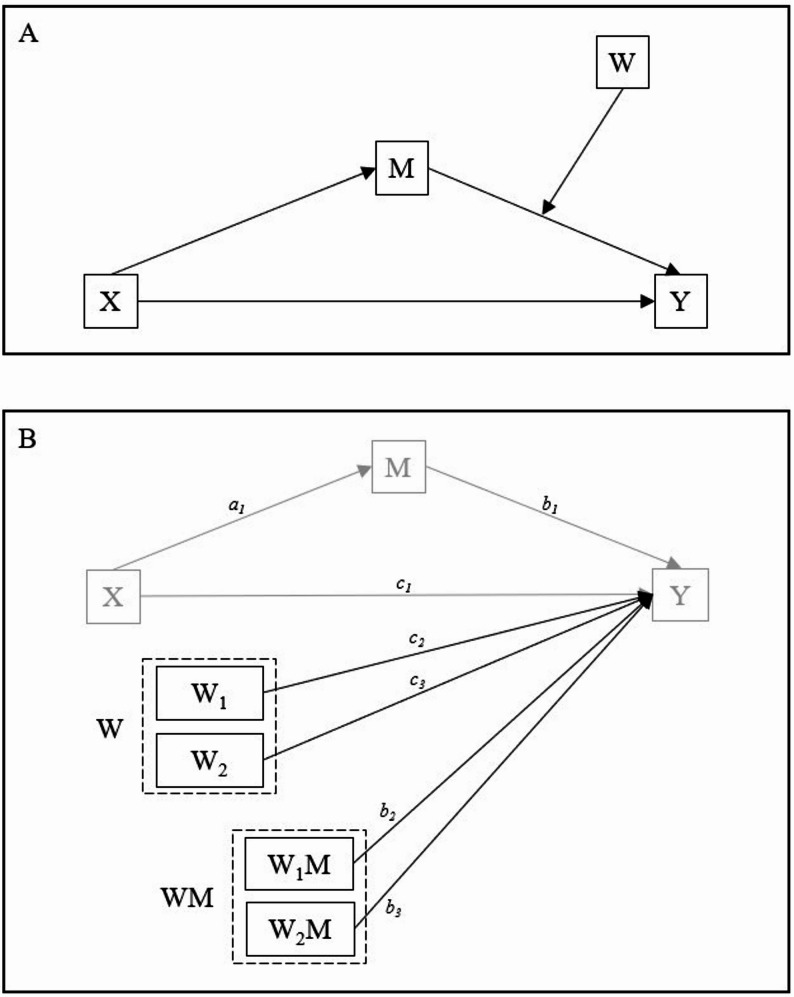

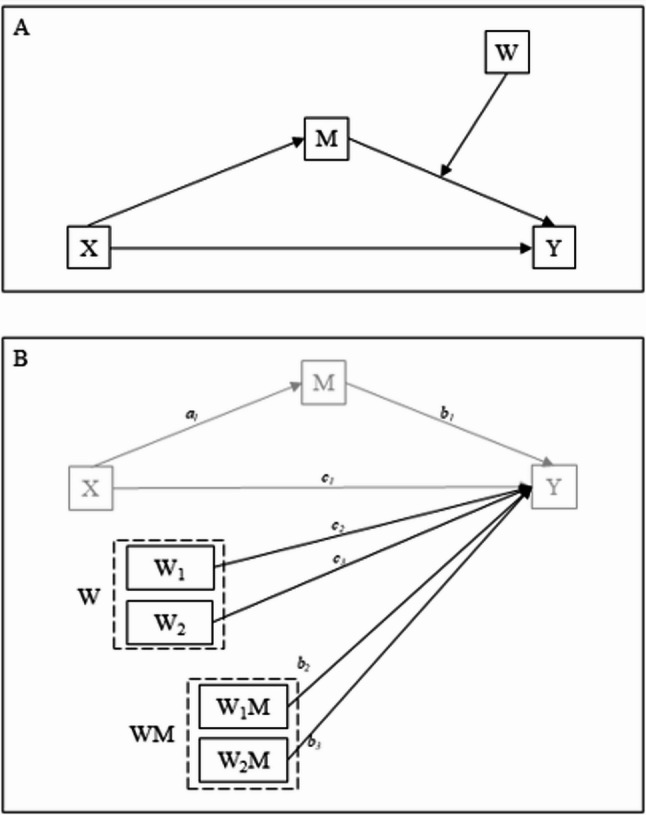
Figure 1 A conceptual (panel A) and statistical (panel B) diagram of the moderated mediation model, consisting of a simple mediation model in which the indirect effect is moderated by a common mediator. Abbreviations: X = independent variable (MEQ); M = mediator (PSQI); Y = dependent variable (SF-12); W = moderator (GSL-TPAQ); a1 = direct effect of X on M; b1 = direct effect of M on Y; b2 and b3 = interaction effects of W and M on Y; c1 = direct effect of X on Y; c2 and c3 = direct effect of W on Y

Third, moderated mediation was tested using Model 14 of the PROCESS macro (Fig. 1A), which evaluates whether the mediation effect of a variable (M) on the relationship between an independent variable (X) and a dependent variable (Y) is influenced by a moderator (W). The model included four variables: (i) MEQ score as the independent variable (X), (ii) SF-12 score as the dependent variable (Y), (iii) PSQI score as the mediator (M), and (iv) physical activity (GSL-TPAQ categorization) as the moderator (W) of the M→Y path. Physical activity was entered as a categorical moderator with three levels, coded using dummy variables: low activity served as the reference category, while medium and high activity levels were coded as W1 and W2, respectively. The model structure, as illustrated in Fig. 1B, includes direct effects (c_1_, c_2_, c_3_), indirect effects (a_1_, b_1_), a total indirect effect (ab), and interaction terms (b_2_ for W_1_M, b_3_ for W_2_M). Additionally, the PROCESS macro provides conditional indirect effects, which estimate how the relationship between X and Y, mediated by M, varies as a function of W. The model also includes an index of moderated mediation (the difference between the conditional indirect effects), the significance of which indicates whether the indirect effect is significantly moderated by W. For the last two variables, the software does not provide *p*-values, and the significance is traceable only in the absence of 0 in the CIs.

For clarity in the results section, we refer to the “first path” as the regression estimating the effect of chronotype (X) on sleep quality (M, a_1_), and the “second path” as the set of regressions estimating the direct effects of X (c_1_), M (b_1_), W (c_2_, c_3_), and the interaction terms (b_2_, b_3_) on Y.

## Results

### Description of the sample

The sample consisted of 691 subjects, with descriptive data presented in Table 1. The sample was predominantly comprised of males, normal-weight and second-year students, with scientific diploma and coming from the regional area of Lombardy. The higher proportion of male representation was consistent with the overall gender distribution of students in the School of Sports Science at the University of Milan, as previously reported in our publications on other samples drawn from the same population [24, 25].

**Table 1:** Descriptive statistics of the sample

Variable	Values
Age (years), mean ± SD	20.11 ± 1.22
Male n (%)	506 (73.2%)
Female (n (%))	185 (26.8%)
BMI (kg/m^2^), mean ± SD	22.27 ± 2.46
Underweight n (%)	27 (3.9%)
Normal weight n (%)	573 (82.9%)
Overweight n (%)	91 (13.2%)
Enrollment year	
Second year n (%)	417 (60.35%)
Third year n (%)	274 (39.65%)
Education level	
Classical Diploma	22 (3.18%)
Language Diploma	44 (6.37%)
Scientific Diploma	400 (57.88%)
Teaching Diploma	36 (5.21%)
Technical Diploma	145 (20.98%)
Vocational Diploma	44 (6.37%)
Origin	
Urban area	109 (15.78%)
Provincial area	211 (30.54%)
Regional area	312 (45.17%)
Outside the region	59 (8.54%)

*Abbreviations*: *BMI* body mass index

Questionnaire results are reported in Table 2. The majority of the sample showed intermediate chronotype preferences and good sleep quality.

**Table 2:** Questionnaire results

Variable	Values
GSL-TPAQ score (LSI), mean ± SD	62.29 ± 27.69
MEQ score (a.u.), mean ± SD	50.63 ± 8.22
E-types n (%)	89 (13%)
N-types n (%)	491 (71.1%)
M-types n (%)	111 (16.1%)
PSQI score (a.u.), mean ± SD	4.62 ± 2.16
Bad sleepers n (%)	222 (32.1%)
Good sleepers n (%)	469 (67.9%)
SF-12	
PCS (a.u.), mean ± SD	51.77 ± 5.65
MCS (a.u.), mean ± SD	41.73 ± 9.65

*Abbreviations*: *GSL-TPAQ* Godin-Shepard Leisure-Time Physical Activity Questionnaire, *LSI* Leisure Score Index, *a.u.* arbitrary unit, *MEQ* Morningness-Eveningness Questionnaire, *PSQI* Pittsburgh Sleep Quality Index, *SF-12* Short-Form Health Survey, *PCS* Physical Component Summary, *MCS* Mental Component Summary

### Three-way ANCOVA

**Fig. 2:**
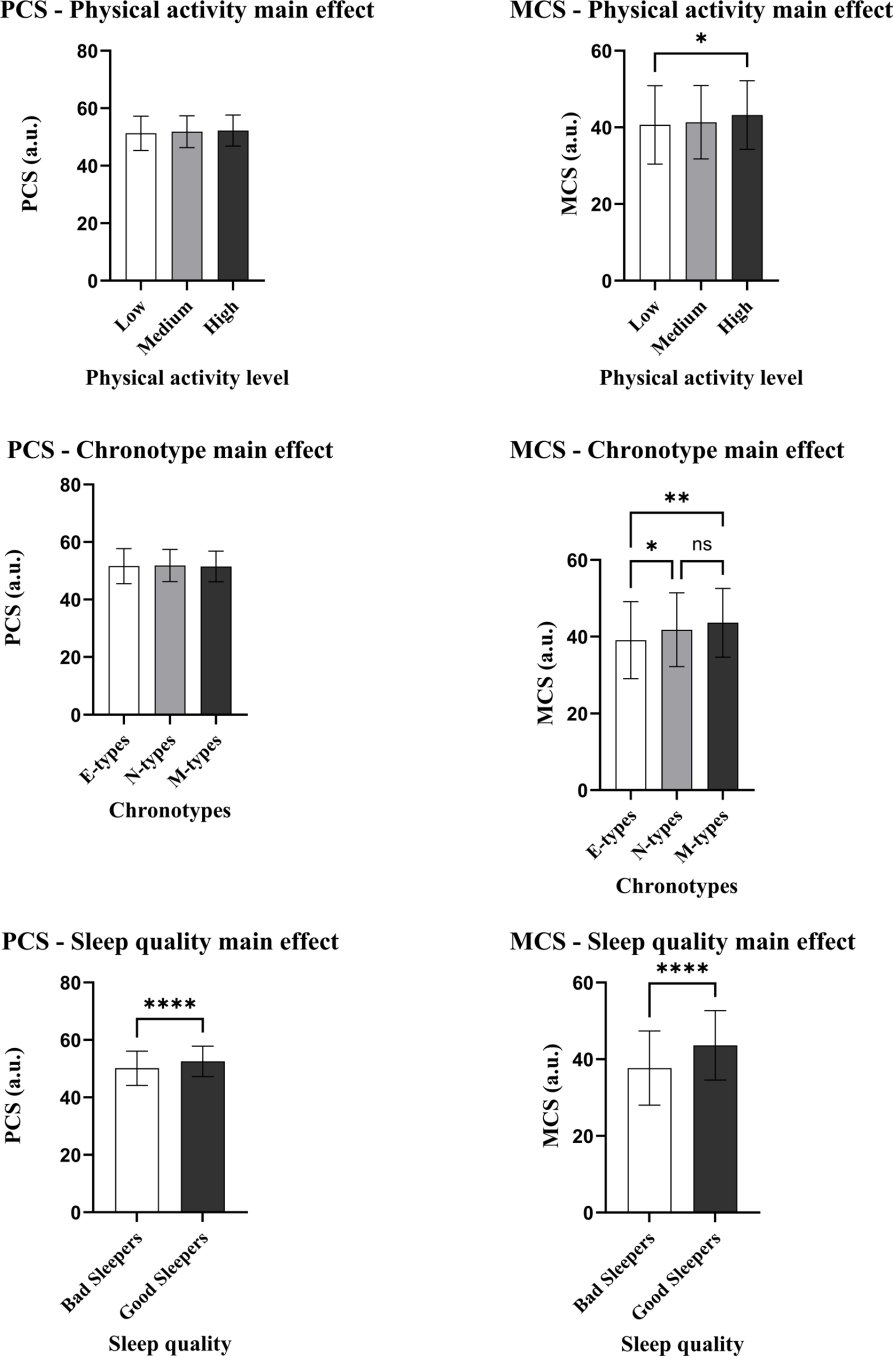

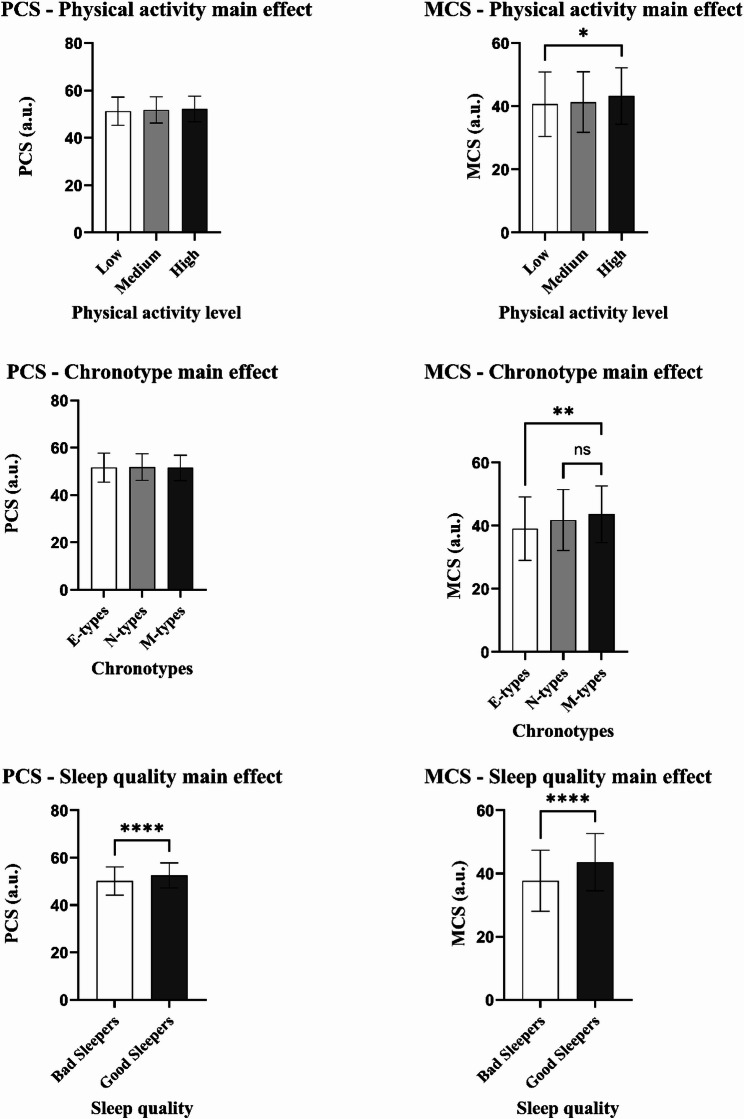
Main effects of physical activity level, chronotype, and sleep quality on physical (PCS) and mental health (MCS). Abbreviations: PCS = Physical Component Summary; MCS = Mental Component Summary. Superscripts: * = p ≤ 0.05; ** = p < 0.01; *** = p < 0.001

The mean and standard deviations for PCS and MCS, stratified by physical activity, chronotype, and sleep quality categorizations, are reported in Table 3; Fig. 2.

**Table 3:** Mean ± SD of physical and mental health (PCS and MCS) stratified for physical activity level, chronotype and sleep quality categorization

ANCOVA main effects	PCS (a.u.) mean ± SD	MCS (a.u.) mean ± SD
Physical activity		
Low	51.28 ± 5.98	40.65 ± 10.21^b^
Medium	51.82 ± 5.52	41.35 ± 9.56
High	52.24 ± 5.18	43.23 ± 8.97 ^b^
Chronotype		
E-types	51.61 ± 6.10	39.08 ± 10.05^c, d^
N-types	50.86 ± 5.60	41.79 ± 9.64^c^
M-types	51.49 ± 5.33	43.6 ± 9.00^d^
Sleep quality		
Bad sleepers	50.14 ± 5.97^a^	37.7 ± 9.68^e^
Good sleepers	52.55 ± 5.32^a^	43.64 ± 9.04 ^e^

*Abbreviations:* *PCS* Physical Component Summary, *MCS* Mental Component Summary, *a.u.* arbitrary unit

Superscript ^a^ = *p* < 0.001; ^b^ *= p* = 0.05; ^c^ = *p* = 0.043; ^d^ = *p* = 0.007; ^e^ = *p* < 0.001

The three-way ANCOVA analysis for PCS was statistically significant only for the sleep quality main effect (*F*_(1, 690)_ = 17.15, *p* < 0.001, *ƞ*_*p*_^*2*^ = 0.03), with good sleepers reporting higher PCS scores than bad sleepers. Therefore, individuals who reported good sleep had better physical health than those who reported bad sleep quality. Physical activity (*F*_(2, 689)_ = 0.47, *p* = 0.62, *ƞ*_*p*_^*2*^ = 0.001) and chronotype (*F*_(2, 689)_ = 0.06, *p* = 0.94, *ƞ*_*p*_^*2*^ < 0.001) main effects were not statistically significant, as well as the four interactions (physical activity * chronotype = *F*_(4, 687)_ = 0.26, *p* = 0.9, *ƞ*_*p*_^*2*^ = 0.002; physical activity * sleep = *F*_(2, 689)_ = 0.22, *p* = 0.8, *ƞ*_*p*_^*2*^ = 0.001; sleep * chronotype = *F*_(4, 687)_ = 0.26, *p* = 0.9, *ƞ*_*p*_^*2*^ = 0.002; physical activity * chronotype * sleep = *F*_(4, 687)_ = 0.61, *p* = 0.66, *ƞ*_*p*_^*2*^ = 0.004).

Regarding MCS, the three-way ANCOVA analysis revealed that all the three main effects were statistically significant (physical activity main effect: *F*_(2, 689)_ = 2.94, *p* = 0.05, *ƞ*_*p*_^*2*^ = 0.009; chronotype main effect: *F*_(2, 689)_ = 4.91, *p* = 0.008, *ƞ*_*p*_^*2*^ = 0.014; sleep quality main effect: *F*_(1, 690)_ = 15.84, *p* < 0.001, *ƞ*_*p*_^*2*^ = 0.023). Low physical activity tertile reported a significantly lower (worse) MCS than the high (*p* = 0.05); E-types collected the lowest MCS compared to M-types (*p* = 0.007) and N-types (*p* = 0.043); finally, bad sleepers showed lower MCS than good sleepers (*p* < 0.001). Thus, participants with the highest physical activity level, morning chronotype and good sleep quality reported the highest mental health. The four interactions showed no statistically significant differences (physical activity * chronotype = *F*_(4, 687)_ = 0.26, *p* = 0.9, *ƞ*_*p*_^*2*^ = 0.002; physical activity * sleep = *F*_(2, 689)_ = 0.22, *p* = 0.8, *ƞ*_*p*_^*2*^ = 0.001; sleep * chronotype = *F*_(4, 687)_ = 0.26, *p* = 0.9, *ƞ*_*p*_^*2*^ = 0.002; physical activity * chronotype * sleep = *F*_(4, 687)_ = 0.61, *p* = 0.66, *ƞ*_*p*_^*2*^ = 0.004).

### Correlation analysis

Correlation analysis results are reported in Table 4.

**Table 4:** Correlation results between physical health (PCS) and mental health (MCS)

Variable	PCS	MCS
MEQ score	*r*_*p*_ = 0.02 *p* = 0.72	***r***_***p***_ **= 0.14** ***p*** ***<*** **0.001**
PSQI score	***r*** _***p***_ **=** **− 0.21** ***p*** ***<*** **0.001**	***r***_***p***_ **=** **−** **0.38** ***p*** ***<*** **0.001**

*Abbreviations*: *PCS* Physical Component Summary, *MCS* Mental Component Summary, *MEQ* Morningness-Eveningness Questionnaire, *PSQI* Pittsburgh Sleep Quality Index

Bold indicates statistically significant effects (p < 0.05)

 MEQ scores showed no significant correlation with PCS, whereas PSQI scores were negatively correlated with PCS. Given these results, testing the moderated mediation model for PCS has not been possible.

MCS correlated directly with MEQ score and inversely with PSQI score, meaning that mental health improved with higher MEQ scores (indicating morningness) and lower PSQI scores (indicating good sleep quality). Moreover, the MEQ score correlated inversely with PSQI scores (*r*_*p*_ = -19, *p* < 0.001), suggesting that sleep quality improved with higher MEQ scores. Based on these results, it has been possible to perform the moderated mediation model for MCS.

### Moderated mediation model

Table 5 presents the results of the moderated mediation model predicting MCS scores. The first path from chronotype (MEQ score) to sleep quality (PSQI) was statistically significant (*p* < 0.01), with a negative coefficient indicating that individuals with greater morningness tend to report better sleep quality. The sex covariate was significant, with a negative coefficient, indicating that males reported lower PSQI scores, and therefore better sleep quality.

**Table 5:** Moderated mediation results of MEQ score, PSQI score and physical activity levels on mental health (MCS)

MCS				
	Coeffcient	SE	95% CI Lower limit	95% CI Upper limit
*Outcome: PSQI score* *R* = 0.26 *R*^*2*^ = 0.7 *F*_(3, 687)_ = 17.20, *p* < 0.001				
MEQ score (a_1_)	**−0.5**	**0.01**	**−0.7**	**−0.3**
Age	0.12	0.07	−0.1	0.25
Sex	**−0.91**	**0.18**	**−1.26**	**−0.56**
*Outcome: MCS* *R* = 0.44 *R*^*2*^ = 0.19 *F*_(8, 682)_ = 20.39, *p* < 0.001				
MEQ score (c_1_)	0.07	0.04	-0.1	0.16
PSQI (b_1_)	**−2.19**	**0.28**	**−2.73**	**−1.64**
W1 – medium (a_2_)	−2.68	1.96	−6.54	1.18
W2 – high (a_3_)	−1.53	1.93	−5.32	2.27
PSQI * W1 – medium (b_2_)	**0.8**	**0.38**	**0.05**	**1.54**
PSQI * W2 – high (b_3_)	**0.8**	**0.38**	**0.04**	**1.55**
Age	−0.41	0.27	−0.95	0.13
Sex	**2.41**	**0.78**	**0.88**	**3.93**
*Conditional indirect effect*				
W0 – low	**0.11**	**0.03**	**−0.06**	**0.17**
W1 – medium	**0.07**	**0.02**	**0.04**	**0.11**
W2 – high	**0.07**	**0.02**	**0.03**	**0.12**
*Index of moderated mediation*				
W1 – medium	**−0.04**	**0.02**	**−0.08**	**−0.01**
W2 – high	**−0.04**	**0.02**	**−0.07**	**−0.01**

*Abbreviations*: *MCS* Mental Component Summary, *MEQ* Morningness-Eveningness Questionnaire, *PSQI* Pittsburgh Sleep Quality Index, *W *mediator

Bold indicates statistically significant effects (p < 0.05)

The second path was also statistically significant. Sleep quality, in turn, significantly predicted mental health (MCS) (*p* < 0.01), with lower PSQI scores (i.e., better sleep) associated with higher MCS scores. This suggests that sleep quality serves as a mediator of the relationship between chronotype and mental health.

The direct effects of physical activity level on MCS were not statistically significant. However, both interaction terms (PSQI × activity level) were significant (*p* = 0.04), indicating that physical activity moderates the relationship between sleep and mental health.

The analysis of conditional indirect effects revealed that the mediating role of sleep quality (PSQI) in the relationship between chronotype (MEQ score) and mental health (MCS score) varied depending on physical activity levels. The indirect effect was statistically significant across all activity groups, but differed in magnitude. Among individuals with low physical activity, the indirect effect was strongest (Effect = 0.11, 95% CI [0.06, 0.17]), indicating that bad sleep more substantially mediates the impact of chronotype on mental health in this group. In contrast, the effect was weaker among those with medium (Effect = 0.07, 95% CI [0.04, 0.11]) and high levels of physical activity (Effect = 0.07, 95% CI [0.03, 0.12]).

The index of moderated mediation was statistically significant for either the high or the medium activity groups, reinforcing data from the conditional indirect effects and indicating that the strength of the indirect effect of chronotype on mental health via sleep quality is significantly weaker among individuals with medium and high physical activity compared to those with low activity levels. The sex covariate was significant and positive, indicating that males reported higher MCS scores, and therefore better mental health.

**Fig. 3:**
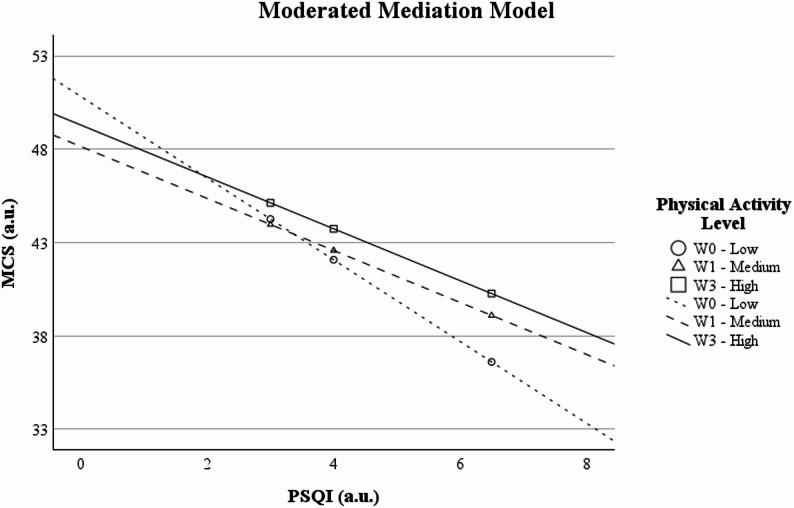
Moderated mediation model illustrating the mediating effect of PSQI score on mental health (MCS), moderated by physical activity level. Abbreviations: MCS = Mental Component Summary; PSQI = Pittsburgh Sleep Quality Index. Symbols: ○ = 16th, 50th, 84th percentile of the low physical activity level; Δ = 16th, 50th, 84th percentile of the medium physical activity level; □ = 16th, 50th, 84th percentile of the high physical activity level

In Fig. 3, the lines show that, for the same level of sleep quality, people with medium and higher levels of physical activity tend to have slightly higher mental health scores. The slope of the line is steeper in the low-activity group, suggesting that sleep has a greater impact on mental health for those who engage in little physical activity.

## Discussion

The current study examined the relationships among physical activity, chronotype, sleep quality, and mental health in a cohort of 691 physically active Italian university students. Although we initially aimed to explore associations with both physical and mental health, the analysis was ultimately restricted to mental health outcomes due to data limitations.

We employed a moderated mediation model to investigate whether the effect of chronotype (MEQ score) on mental health (MCS score) is mediated by sleep quality (PSQI score), and whether this indirect effect is moderated by physical activity level. The results suggest that individuals with a stronger morning preference (higher MEQ scores) tend to report better mental health, a finding consistent with the three-way ANCOVA, which showed that morning types (M-types) had the highest mental health scores.

Notably, sleep quality emerged as a significant mediator: better sleep quality was associated with improved mental health, partially explaining the positive effect of morningness. This mediating role was also supported by the three-way ANCOVA, which showed that good sleepers had significantly better mental health than bad sleepers.

Moreover, physical activity level could moderate this mediation process. Individuals with lower physical activity may be more sensitive to the mediating influence of sleep in the chronotype–mental health relationship. In other words, the indirect effect of chronotype on mental health via sleep quality was strongest among individuals with low physical activity, and weaker among those with medium or high physical activity levels. This pattern suggests that physical activity may play a protective or buffering role in the chronotype–sleep–mental health pathway. In particular, individuals with medium or high activity levels might experience a reduced dependence on sleep quality for maintaining mental health, potentially because regular physical activity enhances psychological resilience or mitigates the effects of bad sleep on mental health.

Physical activity—more precisely, regular physical activity—is a well-established contributor to improved mood, well-being, and relaxation, as well as a protective factor against depression and daily stress [13]. The mechanisms through which physical activity exerts its effects on mental health are multifaceted. These include the enhancement of neuroplasticity, modulation of the endocrine system, improvements in self-esteem and self-efficacy, and the regulation of inflammation and oxidative stress [46–48]. Notably, physical activity has been shown to be as effective as pharmacological or psychological treatments in alleviating symptoms of anxiety, psychological distress, and depression, primarily through activation of the serotonergic and noradrenergic systems [49–52].

Given this evidence, researchers are increasingly implementing physical activity protocols and interventions aimed at reducing mental distress among university students [53, 54]. Our findings support the hypothesis that not only regular, but also sustained engagement in physical activity can improve mental health. Indeed, among participants with an LSI score above 49, the moderated mediation effect was weaker. A score of 49 corresponds approximately to engaging in vigorous physical activity five times a week for at least 15 min per session, or nearly ten sessions of moderate-intensity physical activity of the same duration. Furthermore, our data suggest that in highly physically active individuals, the beneficial effects of exercise may partially bypass or buffer the negative impact of bad sleep quality on mental well-being.

Sleep quality is another essential factor in promoting mental health. Dickinson and colleagues (2017) reinforce our results and mediation model, reporting that sleepiness mediates the relationship between eveningness (E-types) and both anxiety and depression in young adults [34]. A recent meta-analysis has supported a causal relationship between sleep and psychological well-being, suggesting that improvements in sleep quality can reduce the severity of mental health symptoms, including stress, depression, and anxiety, particularly in non-clinical populations [55]. Additionally, other studies on university students suggest that improvements in sleep quality are a crucial factor in alleviating stress, anxiety, and mental health [9, 11, 35].

The causal link between sleep and mental health may be explained by difficulties in emotional regulation, such as the amplification of adverse life events, impaired regulation capacity, and increased reliance on maladaptive emotion regulation strategies [56–58]. Additionally, another recent review has proposed that the connection between sleep and conditions such as depression and anxiety may be mediated by dysregulation of the cholinergic system, altered BDNF (brain-derived neurotrophic factor) secretion, and disruptions in the prefrontal cortex–amygdala circuitry [59].

Sleep quality appears to be influenced by chronotype. In our moderated mediation model, higher MEQ scores—indicating a stronger morningness tendency—were associated with better sleep quality. These results, combined with the findings of the three-way ANCOVA, suggest that M-types generally experience better sleep than E-types. However, the direct effect of chronotype on mental health was not statistically significant, showing only a trend toward significance. This suggests that chronotype does not exert a direct influence on mental health in our sample but may instead affect it indirectly through its impact on sleep quality. Indeed, the moderated mediation analysis confirmed that sleep quality mediates the relationship between chronotype and mental health: individuals with a morning chronotype tend to sleep better, and this better sleep quality is associated with improved mental well-being.

Our findings are partially consistent with previous literature. Several studies conducted in university student populations have reported that M-types tend to have better sleep quality than E-types [8, 60], and are also less likely to experience mental health problems [8, 11]. However, considering broader samples, and in line with findings by Kim and colleagues (2023), our results support a mediation model in which sleep quality serves as a key pathway linking chronotype and mental health [31].

In this context, another variable that was not evaluated in the present study should be considered: eating patterns and meal timing. Indeed, food intake, caffeine consumption, and night eating behaviors can be influenced by chronotype, with E-types typically exhibiting poorer eating habits and a higher tendency toward night eating. Moreover, eating patterns and timing may interact with both sleep quality and mental health, representing an additional factor that could affect university students’ overall health [61, 62].

The moderated mediation analysis also identified sex as a significant covariate. First, in the first path analyzing the relationship between chronotype and sleep quality, females were more likely to report worse sleep quality. This finding is consistent with previous research [14, 63, 64] and may be attributed to women’s greater need for sleep, hormonal fluctuations, and differing physiological responses [63, 65–67]. Second, in the second path of the moderated mediation model, females also reported worse mental health outcomes. This aligns with prior findings indicating that female sex may negatively impact mental health (e.g [68, 69]), potentially due to higher levels of stress, greater emotional burden, or increased vulnerability to hormonal and biological fluctuations [68–71].

Finally, our data did not allow us to perform the analysis for physical health, a limitation also reported in previous studies [11]. This may be attributed to the characteristics of our sample, which generally exhibited high levels of physical activity and good sleep quality. Given that both physical activity and sleep quality are key factors influencing physical health, the low variability in these variables may have limited the ability to detect significant effects [72–74].

The results of the current study should be interpreted in light of its strengths and limitations. Among the strengths are the large sample size, the selection of an inherently active population, and the use of an analytical model that integrates multiple lifestyle factors—elements that are rarely examined together, particularly in relation to their interrelationships. Among the limitations are: the absence of objective measures for physical activity and sleep; the overrepresentation of male participants; the lack of comparison groups with lower levels of physical activity; the use of the GSL-TPAQ which, although employed in several published studies with Italian samples [75–78], has not yet been formally validated; the lack of assessments of dietary habits, behaviors, and times; and the fact that BMI was not included as a main effect in the ANCOVA analysis—being used solely for descriptive purposes—because the predominance of normal-weight participants and the characteristics of highly active populations render BMI a potentially misleading indicator, as it does not distinguish between fat mass and fat-free mass.

## Conclusion

This study highlights the complex interplay between physical activity, chronotype, sleep quality, and mental health in university students. Our findings suggest that morningness is associated with better sleep quality, which in turn supports better mental health. Importantly, the mediating role of sleep appears to be influenced by the level of physical activity: students with medium or high activity levels may experience a reduced negative impact of bad sleep on mental well-being.

Considering the increasing concerns about students’ mental health in recent years and the growing search for non-pharmacological approaches to alleviate psychological distress, these findings underscore the importance of promoting physical activity as a protective strategy, particularly for individuals vulnerable to poor sleep or with an evening chronotype. Poor sleep quality is especially common among university students, who often face the combined challenges of academic, social, and sport-related commitments. In this context, regular, daily, and sustained physical activity may help buffer the adverse effects of inadequate sleep, reinforcing its role as a cost-effective and accessible tool to support mental health in young adult populations.

Finally, future studies should consider evaluating diet, eating patterns, and meal timing, as it has been reported that food intake, caffeine consumption, and night eating behaviors can affect sleep duration and quality, and consequently may have an indirect impact on students’ health.

## Data Availability

The data that support the findings of this study are not openly available and are available from the corresponding author upon reasonable request.

## Abbreviations

ANCOVA: Analysis of Covariance
a.u.: Arbitrary Units
CIs: Confidence Intervals
E-types: Evevening-Types
GSL-TPAQ: Godin-Shepard Leisure-Time Physical Activity Questionnaire
LSI: Leisure Score Index
M: Mediator
MEQ: Morningness-Eveningness Questionnaire
M-types: Morning-Types
MCS: Mental Component Summary
N-types: Neither-Types
PCS: Physical Component Summary
PSQI: Pittsburgh Sleep Quality Index
SD: Standard Deviations
SF-12: Short-Form Health Survey
W: Moderator
X: Independent variable
Y: Dependent variable
